# Supplementary material for: Elementary School Teachers’ Perceptions of COVID-19-Related Restrictions on Food Allergy Management
Source: Nutrients. 2022 Jun 29;14(13):2714. doi: 10.3390/nu14132714 (PMC9268574; doi:10.3390/nu14132714)
Supplement: Supplementary file 1 [file nutrients-14-02714-s001.zip › nutrients-1765075-supplementary.pdf]

Supplementary Table S1. Interview Guide for Teachers

1. Introductory question
   - Would you please tell us a bit about your role and background?
   - Before becoming a teacher, how much did you know about food allergy?
   - Since becoming a teacher, what are your experiences with food allergy?
     - Where did you learn about food allergy?

2. Content questions
   - How does your school manage food allergy?
   - Please describe your confidence in managing food allergy in the classroom.
   - What kinds of supports, training and/or resources do you currently have to manage a severe allergic reaction in your classroom?
   - What additional kinds of supports, training and/or resources would you need to manage a severe allergic reaction in your classroom?
   - If you ever had an experience working or managing a child with food allergy, please discuss.
   - Research tells us that children with food allergy are often bullied or teased because of their food allergy. If you've ever seen this, please share your experience and ability/ comfort in being able to handle the situation.
   - How do you, as a teacher, feel about being responsible for a food allergic child?
   - The COVID-19 pandemic has changed every aspect of our lives. As teachers you have been responsible for your students' well-beings in ways, we did not imagine two years ago. Please describe any differences in how food allergy is managed in your classroom/ school during the pandemic to before the pandemic.

3. Closing questions
   - Of all the topics we discussed today, what is the most important part for you?
   - Now, I will provide a brief summary about what we talked about. Please correct me if anything is incorrect. (Provide summary)
     - How was that?
     - Did I capture what you said or did I make some mistakes? (Correct if necessary)
   - We've talked a lot today about working with a child with food allergy. Is there anything that I should have asked, but did not?
     - Would you like to add anything else?
